# Nitrogen Removal From Nitrate-Containing Wastewaters in Hydrogen-Based Membrane Biofilm Reactors *via* Hydrogen Autotrophic Denitrification: Biofilm Structure, Microbial Community and Optimization Strategies

**DOI:** 10.3389/fmicb.2022.924084

**Published:** 2022-06-02

**Authors:** Kun Dong, Xinghui Feng, Yi Yao, Zongqiang Zhu, Hua Lin, Xuehong Zhang, Dunqiu Wang, Haixiang Li

**Affiliations:** ^1^College of Environmental Science and Engineering, Guilin University of Technology, Guilin, China; ^2^The Guangxi Key Laboratory of Theory and Technology for Environmental Pollution Control, Guilin, China

**Keywords:** response surface methodology, microbial community, characterization, calcification, MBFR

## Abstract

The hydrogen-based membrane biofilm reactor (MBfR) has been widely applied in nitrate removal from wastewater, while the erratic fluctuation of treatment efficiency is in consequence of unstable operation parameters. In this study, hydrogen pressure, pH, and biofilm thickness were optimized as the key controlling parameters to operate MBfR. The results of 653.31 μm in biofilm thickness, 0.05 MPa in hydrogen pressure and pH in 7.78 suggesting high-efficiency ${\mathrm{NO}}_{3}^{-}-N$ removal and the ${\mathrm{NO}}_{3}^{-}-N$ removal flux was 1.15 g·m^−2^ d^−1^. 16S rRNA gene analysis revealed that *Pseudomonas*, *Methyloversatilis*, *Thauera*, *Nitrospira*, and *Hydrogenophaga* were the five most abundant bacterial genera in MBfRs after optimization. Moreover, significant increases of *Pseudomonas* relative abundances from 0.36 to 9.77% suggested that optimization could effectively remove nitrogen from MBfRs. Membrane pores and surfaces exhibited varying degrees of calcification during stable operation, as evinced by Ca^2+^ precipitation adhering to MBfR membrane surfaces based on scanning electron microscopy (SEM), atomic force microscopy (AFM) analyses. Scanning electron microscopy–energy dispersive spectrometer (SEM–EDS) analyses also confirmed that the primary elemental composition of polyvinyl chloride (PVC) membrane surfaces after response surface methodology (RSM) optimization comprised Ca, O, C, P, and Fe. Further, X-ray diffraction (XRD) analyses indicated the formation of Ca_5_F(PO_4_)_3_ geometry during the stable operation phase.

## Introduction

Abundant toxic and harmful pollutants have been discharged into natural environments in recent years, leading to significant deterioration in groundwater and surface water quality (Liu et al., 2022). Nitrate is mobile in water and soil, with excessive nitrate from sewage, agricultural fertilizers, or intensive agriculture easily entering groundwater and surface water systems, where it can cause greater harm to environments. Indeed, due to excessive eutrophication nitrate has become a serious environmental problem in most regions of the world, and in China, owing to the discharge of municipal wastewater. Nitrate cannot be easily removed by traditional denitrification technology in water purification plants. Rather, several physical and chemical techniques are used for nitrate in groundwater and surface water including ion exchange, chemical reduction, physical adsorption, and reverse osmosis (Della Rocca et al., 2007; Demirel and Bayhan, 2013). However, these physical and chemical methods are generally characterized by serious secondary pollution, high costs, and complex follow-up treatments, thereby considerably limiting their practical application. Consequently, the need to identify more efficient and green biological treatment technologies for nitrate removal has grown in recent years.

Membrane biofilm reactor treatment is a newly developed method for the efficient removal of nitrate from groundwaters and surface waters. MBfR operates under low carbon source and low oxygen availability conditions and combines microporous hollow fiber membrane diffusion aeration with autotrophic biofilm technology to remove oxidizing pollutants from water using hydrogen as a clean and inexpensive electron donor (Xia et al., 2016; Zhou et al., 2016). In MBfRs, microporous membranes act both as a channel for the transmission of hydrogen and as a carrier for microbial attachment, with electron donors (e.g., H_2_) or electron acceptors (e.g., O_2_) delivered to microorganisms living on membrane walls (Rittmann, 2011; Tang et al., 2012). H_2_ diffuses from inside of the membrane to provide bubble-free hydrogen gas to the autotrophic microorganisms. Microbial respiration activity then oxidizes pollutants as electron acceptors and converts them into harmless byproducts. Importantly, hydrogen autotrophic microorganisms can use inorganic carbon as a carbon source and hydrogen as an electron donor to reduce oxidizing substances, thereby conserving energy. Hydrogen is a clean, non-toxic autotrophic denitrifying electron donor. MBfR using H_2_ for denitrification leads to a very good hydrogen utilization efficiency (over 99%) and avoids risks from reactor explosion owing to hydrogen accumulation (Long et al., 2020). Moreover, the development of hydrogen production technology in recent years has led to reduced hydrogen production costs, rendering MBfR a promising, clean, and inexpensive electron donor strategy.

A number of MBfRs have been developed for contaminants such as nitrate, sulfate, bromate, perchlorate, and chromate (Chung et al., 2006; Van Ginkel et al., 2008; Li et al., 2017). In the case of hydrogen as an electron donor, the stoichiometries (Xia et al., 2010) in which ${\mathrm{NO}}_{3}^{-}-N$ is reduced to nitrite and eventually to N_2_ are shown by Eqs. (a), (b):


(a) $${\mathrm{NO}}_{3}^{-}+H_{2}\to {\mathrm{NO}}_{2}^{-}+H_{2}O$$

(b) $$2{\mathrm{NO}}_{2}^{-}+2H^{+}+3H_{2}\to N_{2}+4H_{2}O$$

Nitrate reduction is clearly the source of alkaliniy in MBfRs *via* the consumption of 2 H^+^ equivalents per N equivalent of ${\mathrm{NO}}_{3}^{-}$ , as shown in Eqs. (a), (b). Bicarbonate is added as an inorganic carbon source along with a combination of phosphate solutions (Na_2_HPO_4_ and KH_2_PO_4_) to the feed water to control pH in MBfRs.

Response surface methodology (RSM) is a statistical, mathematical modeling tool that can be used for reactor optimization. The Box–Behnken design (BBD), a kind of RSM design, has been used to investigate optimal levels of key factors for the removal rate of nitrate. During modeling, the system response ( ${\mathrm{NO}}_{3}^{-}-N$ removal rate) is firstly expressed as a function of several factors (pH concentration, hydrogen pressure, and biofilm thickness) and the system function relationship is expressed using reasonable experimental methods and graphical techniques to construct a suitable response surface model of the influencing factors of MBfR hydrogen autotrophic denitrification, followed by the use of RSM.

Calcification is also observed (e.g., *via* precipitates containing Ca^2+^) on the surface of polyvinyl chloride (PVC) hollow fiber membranes during stable operation of MBfRs. Inorganic compounds such as CaCO_3_ undergo precipitation reactions in dynamic equilibrium with MBfRs, wherein the hardness cations present in the MBfR combine with typical anions to form precipitates. Hardness ion precipitates in MBfRs include CaCO_3_, CaHPO_4_, Ca(H_2_PO_4_)_2_, Ca_5_(PO_4_)_3_OH, and Ca_3_(PO_4_)_2_ (Tang et al., 2011). In addition, the precipitation of Ca^2+^ during denitrification and the long-term accumulation of CaCO_3_ (comprising up to 25% of biofilm mass) outside the biofilm form a mineral solid buildup inside the biofilm, thereby increasing mass transfer resistance at the bottom of the biofilm. These dynamics then result in substrates not being able to freely diffuse inside the biofilm and even clogging the device, making the hollow fiber membrane filaments more brittle and leading to long-term negative impacts on the efficient operation of MBfRs (Lee and Rittmann, 2003; Hou et al., 2019). Increased Ca^2+^ on the hollow fiber membrane surface also leads to decreased hydrogen transfer efficiency and nitrogen removal performance in MBfRs. Three factors are related to calcification (i.e., the precipitation of Ca^2+^) on the hollow fiber membrane surface and have been selected for optimization of the reactor to obtain stable and efficient denitrification performances: pH, hydrogen pressure, and biofilm thickness. Hydrogen autotrophic denitrification leads to increased pH, and Ca^2+^ precipitation is closely related to the solution pH. When Na_2_HPO_4_ and KH_2_PO_4_ are used as buffer solutions, the reactor solutions cannot effectively be controlled below pH 8.0, and the dynamic balance of free CO_2_, HCO_3_-, and ${CO}_{3}^{2-}$ in solution directly affects the pH, thereby the precipitation and dissolution balance of Ca^2+^. pH also plays a key role in the precipitation and dissolution equilibrium of Ca^2+^. Hydrogen as an electron donor is an important influencing factor for nitrate and nitrite removal. Insufficient hydrogen (i.e., low hydrogen pressures) during MBfR operation inhibit denitrification, while the supply mode at one end of the MBfR leads to low H_2_ diffusion efficiency from the supply end due to insufficient hydrogen pressure, resulting in insufficient effective control of the pH in the biofilm at distant locations, leading to Ca^2+^ precipitation. The low solubility of hydrogen can also lead to excessive hydrogen, wherein it cannot be fully utilized by the biofilm, resulting in wasted resources. Biofilm thickness is the main factor affecting substrate transfer into the biofilm and pollutant removal efficiency, while biofilm thickness is closely related to Ca^2+^ precipitation. During MBfR operation when increasing calcium precipitation occurred on the biofilm surface, biofilm thickness increased, which led to limited substrate transfer and decreased pollutant removal efficiency.

In this study, PVC hollow fiber membranes were used as biofilm carriers in the reactor. RSM was then used to optimize the operating parameters of MBfR-hydrogen autotrophic denitrification. Calcification on the PVC hollow fiber membrane during the stable operation stage was characterized by scanning electron microscopy (SEM), X-ray diffraction (XRD), scanning electron microscopy–energy dispersive spectrometer (SEM–EDS), X-ray photoelectron spectroscopy (XPS), and atomic force microscopy (AFM). High-throughput sequencing was also used to assess the compositions of microbial communities. Optimal conditions were then investigated through RSM based on the BBD under different conditions. The above investigations were used to explore the operating parameters and surface morphological characteristics of hollow fiber membranes suitable for MBfR-hydrogen autotrophic denitrification, discuss the relationship between biofilm formation and calcification and provide a theoretical basis for reactor operation and control of membrane calcification.

## Materials and Methods

### Experimental Set-Up and Control Strategy

**Figure 1:**
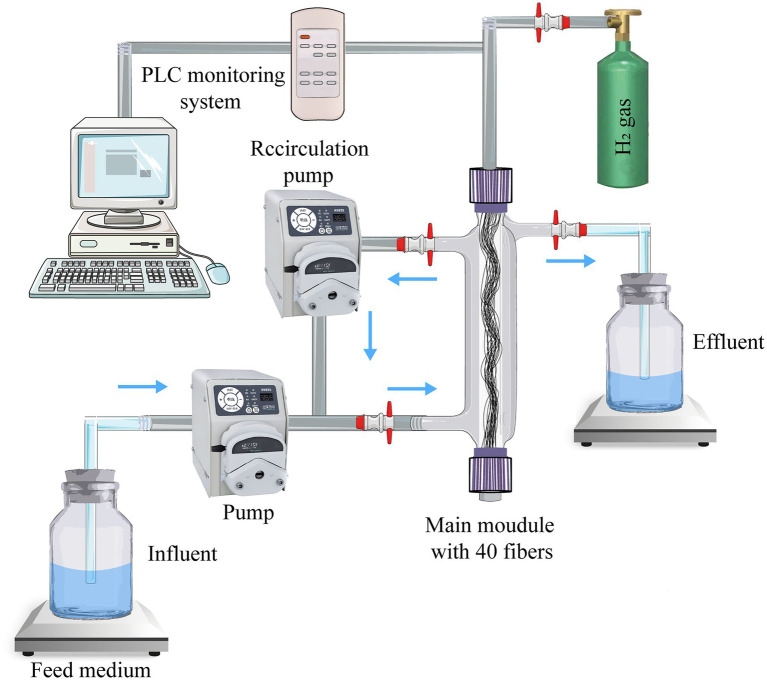
Schematic of the hydrogen-based membrane biofilm reactor (MBfR) used to investigate nitrate reduction.

Detailed MBfR parameters are listed in Table 1 and Figure 1. A single peristaltic pump (BT101L-DG-1, Lead Fluid, Baoding, China) was used to control the nitrate-medium-feed influent rate at 0–3 ml/min. Synthetic domestication water followed a previously described recipe (Xia et al., 2010) and comprised (per liter): 0.128 g of KH_2_PO_4_, 0.434 g of NaHPO_4_, 0.2 g of MgSO_4_·7H_2_O, 0.001 g of CaCl_2_·2H_2_O, 0.001 g of FeSO_4_·7H_2_O, 0.252 g of NaHCO_3_, and 1 ml of a stock solution containing trace elements. The trace element stock solution contained (per liter): 100 mg of ZnSO_4_·7H_2_O, 30 mg of MnCl_2_^.^4H_2_O, 300 mg of H_3_BO_3_, 0.1 mg of H_3_BO_3_, 200 mg of CoCl_2_·6H_2_O, 10 mg of CuCl_2_·2H_2_O, 10 mg of NiCl_2_·6H_2_O, and 30 mg of Na_2_SeO_3_. The trace element solution was produced in a 25.0 l water distribution tank after purging with N_2_ to remove dissolved oxygen (DO) in the influent and mixing the trace solution well by aeration. High recirculation speed (100 ml/min) and high recirculation rate (nearly 100:1 when the influent rate was 1 ml/min) were used in the experiment. Under these conditions, hydrogen autotrophic microorganisms can accumulate on the hollow fiber membrane faster and simultaneously enable the MBfR to reach a fully mixed state. In addition, pure H_2_ was supplied to the interior of the hollow fiber through an inlet in the top center of the reactor, while the pressure range is controlled from 0.02 to 0.06 MPa. Further, a programmable logic controller (PLC) monitoring system connected to the pH and DO probes enables 24-h real-time monitoring of pH and DO inside the MBfR.

**Table 1:** Physical characteristics of the MBfR system.

Parameter	Units	Value
Membrane material		PVC
Active volume	L	1.8
Number of HFM		40
HFM inner diameter	nm	1.0
HFM outer diameter	nm	1.66
Pure water flux	L/m^2^·h	400

### PVC Hollow Fiber Membrane and Experimental Initiation

The main membrane module contained a bundle of 40 hydrophobic hollow fiber membranes (HFMs) constructed from PVC (Watercode, Guangzhou, China). Smaller pore size HFMs constructed with PVC and a pore size of 0.1 μm were used in the MBfR to deliver bubble-free H_2_ through the HFM walls. The reactor volume was 1.8 l and the initial mixed liquor suspended solids (MLSS) concentration was 2,500 mg/l. Activated sludge was added with a 50 ml syringe, with a total volume of added activated sludge of 10% of the MBfR. The activated sludge consisted of sludge from the Qilidian wastewater plant in Guilin and mature hydrogen autotrophic denitrification sludge that was established in the laboratory, mixed at a ratio of 1:2.6 (50 ml of wastewater plant sludge and 130 ml of laboratory sludge). During the laboratory domestication stage when the influent concentration was 10 mg/l, the ${\mathrm{NO}}_{3}^{-}-N$ removal flux was 0.83 g·m^−2^ d^−1^ after 76 days of domestication and the ${\mathrm{NO}}_{3}^{-}-N$ standardized flux was 0.083 g·m^−2^ d^−1^, leading to a final removal rate of the MBfR reaching 90.32%. A layer of yellow–brown solids was also attached to the surface of the PVC hollow fiber membrane module and a uniformly distributed yellow–brown biofilm was observed. Thus, the MBfR start-up domestication stage was established. After the system was successfully initiated, the response surface was optimized on the basis of single-factor experiments to ensure experimental reliability.

### Biofilm Structural Analysis

${\mathrm{NO}}_{3}^{-}-N$ removal rate is an important parameter for characterizing the performance of MBfR-hydrogen autotrophic denitrification and the ${\mathrm{NO}}_{3}^{-}-N$ concentrations of all samples were measured by using a continuous flow analyzer (SAN^++^, Skalar, Netherlands).

Several characterization analyses of the membrane modules based on PVC hollow fiber membranes were conducted in the stable operation stage by exploring calcification. Surface morphology, agglomeration, microstructure, and chemical composition characteristics of PVC hollow fiber membranes in the stable operation stage were assessed with SEM (MIRA3, Tescan, Czech Republic), and SEM–EDS (AZTEC X-MAXN 80, Oxford, United Kingdom). The SEM was operated with an acceleration voltage of 15 kV, a fixed angle of 35°, and an elemental analysis range of Be^4^ to Cf^98^. XPS (ESCALAB250Xi, ThermoFisher Scientific, United States) with a monochromatic Al Kα source (1486.6 eV) was used to measure the elemental composition and chemical state of the membranes. In addition, crystal structures of the samples were assessed with XRD measurements and an X-ray diffractometer (X’PERT PRO, Panalytical, Holland) with a 2*θ* range of 5°–90° and a scan rate of 10°min^−1^. AFM (DIMENSION ICON, Bruker, United States) exhibited significant advantages by providing nanoscale resolution imaging capacities for studying the microscopic properties of materials and obtaining measurements such as the surface roughness of PVC hollow membrane samples.

MBfR biofilms can be used to identify bacterial species *via* high-throughput sequencing when ${\mathrm{NO}}_{3}^{-}-N$ reductions are taking place. A 3 cm piece of the coupon fiber was cut from identical regions of each MBfR before and after optimization experiments. Biomass samples were then sent to Sangon Biotech Co., Ltd. (Shanghai, China) for microbial structural analysis. The 16S rRNA gene sequence libraries were then prepared using polymerase chain reaction (PCR) with universal primers and amplification of the V4–V5 hypervariable regions of genes. Operational taxonomic units (OTUs) at the 97% nucleotide identity threshold were generated, and taxonomic classification of each OTU representative was conducted at the genus level by using the Ribosomal Database Project Classifier.

### RSM Optimization Design

In this study, ${\mathrm{NO}}_{3}^{-}-N$ removal rate was the target index and a response surface model was constructed to evaluate optimization of this rate. In particular, the response surface method was used to investigate the effects of biofilm thickness (A, 600–700 μm), hydrogen pressure (B, 0.02–0.06 mg/l), and pH (C, 6.5–8.5) on denitrification efficiency *via* the hydrogen autotrophic denitrification process, with the ${\mathrm{NO}}_{3}^{-}-N$ removal rate as the response value. A total of 17 three-parameter experimental designs were conducted to evaluate the single and interactive effects of different conditions on the target responses to improve the denitrification performance of the reactor. A model was then established from the results to identify the best parameters to improve denitrification efficiency of hydrogen autotrophic denitrification. The mathematical relationships among variables (biofilm thickness, hydrogen pressure, and pH) and response values can be described by the following quadratic polynomial (Hellmuth et al., 1995):


(c) $$Y=\beta_{0}+\sum \beta_{i}X_{i}+\sum \beta_{ii}X_{i}^{2}+\sum \beta_{ij}X_{j}\left(i,j=1,2,\ldots ,k\right)$$

where *Y* is the predicted response (i.e., the ${\mathrm{NO}}_{3}^{-}-N$ removal rate), *β_0_* is the intercept term, and *β_i_, β_ii_*, and *β_ij_* are the linear, quadratic, and interaction coefficients of the variables, respectively. *X_i_* and *X_j_* are the coefficients of biofilm thickness, hydrogen pressure, and pH. The validity of the model was then examined significantly *via* the second-order polynomial coefficients of determination (*R*-squared) and the analysis of variance (ANOVA) using the “Design Expert” software package (Version 8.0.6, Stat-Ease Inc., Minneapolis, MN, United States).

## Results and Discussion

### RSM Models for Optimizing MBfR Hydrogen Autotrophic Denitrification

**Figure 2:**
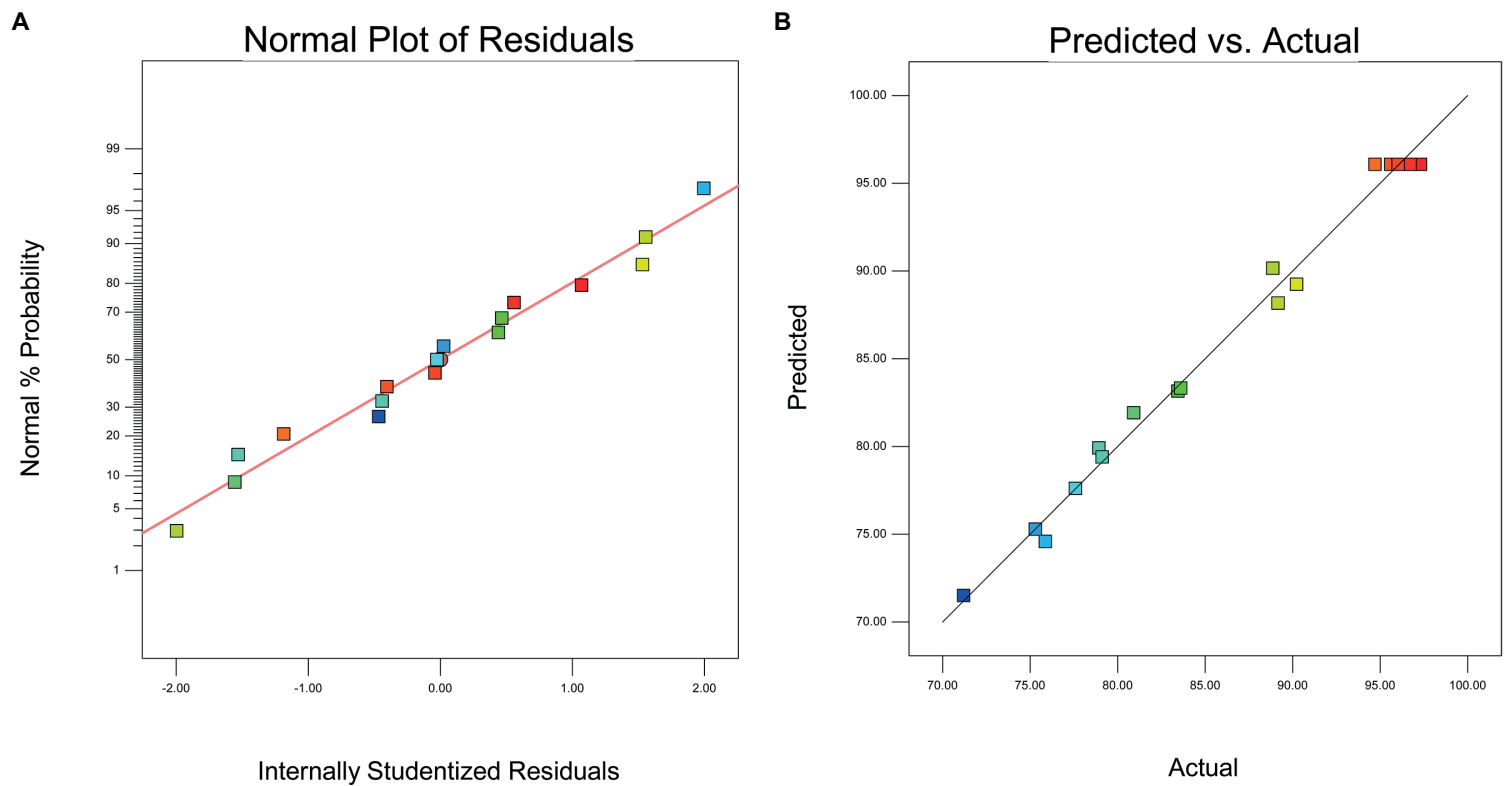
**(A)** Normal plot of residuals and **(B)** plot of predicted vs. actual values.

**Figure 3:**
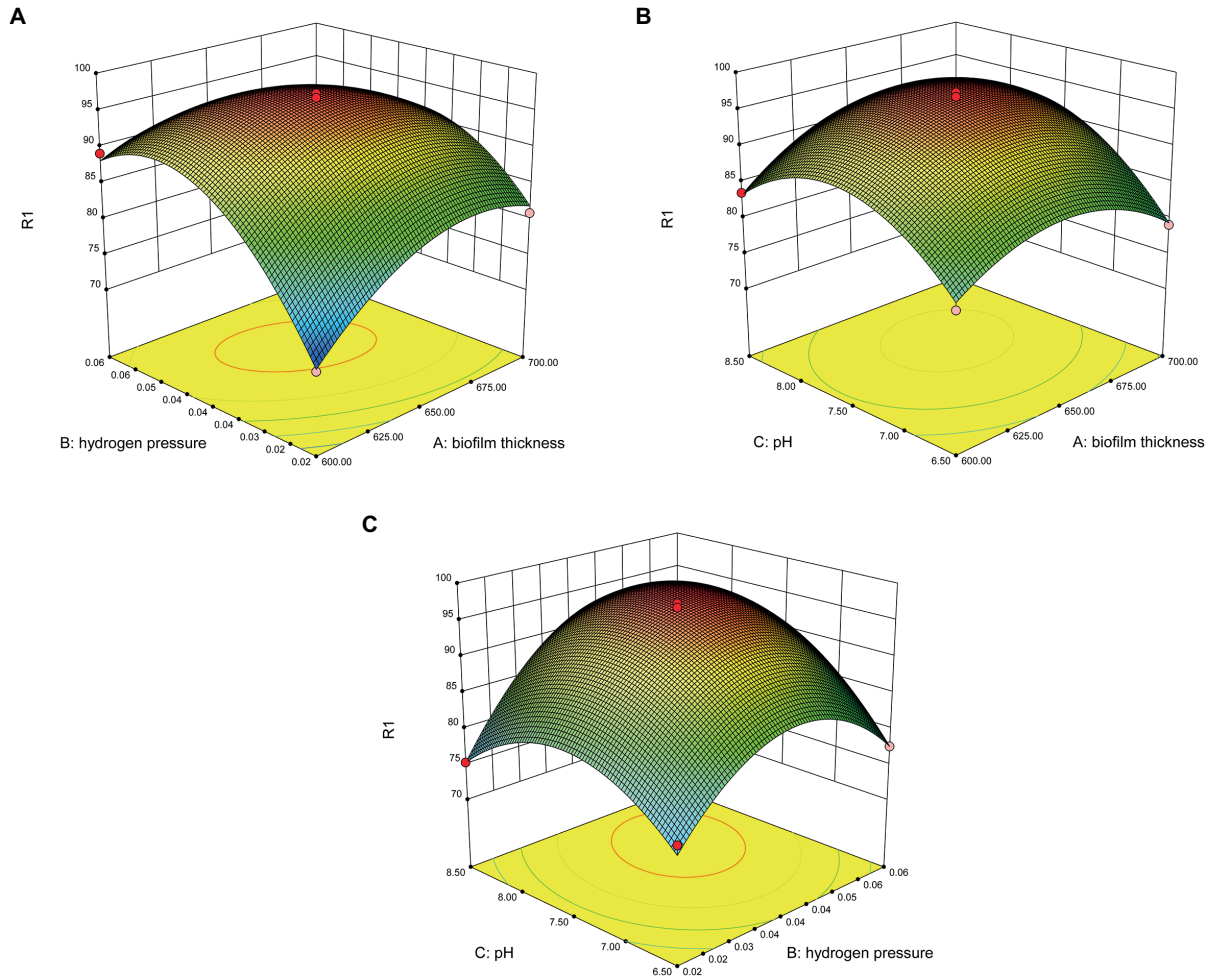
Three-dimensional surface plots for the nitrate removal capacity of MBfR: **(A)** biofilm thickness vs. hydrogen pressure, **(B)** biofilm thickness vs. pH, and **(C)** hydrogen pressure vs. pH.

The interactions of the three reactor components and their optimal levels during the removal of nitrate were further analyzed by using response surface methodology. A normal plot of the residuals for nitrate removal by the MBfR was used to evaluate the reliability of the model (Figure 2A). The internally studentized residuals and normal % probability exhibited primarily linear function relationships and the data points were distributed around linear straight lines with a normal distribution (Figure 2A). The scatter of actual and predicted results for the nitrogen removal capacity of MBfRs based on the three-parameter experiment and RSM model was also evaluated (Figure 2B). Values obtained from the experimental results and those predicted by the model were randomly scattered around the linear straight line, while the experimental and predicted values barely deviated from the straight line, indicating that the error between the actual and predicted values was minimal. Three-dimensional response surface plots provided a visual representation of the relationships between the objective function and the design variable. Specifically, these represent surface derived from the function established by the relationship between two of the three variables mentioned above and the response value, requiring that all other parameters are constant to investigate the primary effects and interactions of the two factors (Polat and Sayan, 2019). The effects of (A) biofilm thickness and hydrogen pressure, (B) biofilm thickness and pH, and (C) hydrogen pressure and pH on the nitrate removal capacity of MBfRs were all evaluated (Figure 3). The ${\mathrm{NO}}_{3}^{-}-N$ removal rate firstly increased and then decreased with increasing hydrogen pressure (from 0.02 to 0.06 MPa) and biofilm thickness (from 600 to 700 μm), respectively, at a specified influent concentration (Figures 3A,B). The mechanism underlying these dynamics was that increasing hydrogen pressure improved the use efficiency of hydrogen as electron donor and the stable concentration of ${\mathrm{NO}}_{3}^{-}-N$ at 0.02 MPa was lower at 0.04 MPa, while the amount of electron donor (H_2_) was insufficient for complete reduction of ${\mathrm{NO}}_{3}^{-}-N$ to N_2_. Thus, continuously increasing hydrogen pressure over a certain range can promote the removal of ${\mathrm{NO}}_{3}^{-}-N$ to a certain extent. However, excessively high hydrogen pressures adversely affected the efficient operation of MBfRs and the removal of nitrate.

Biofilm thickness was also an important factor affecting the removal of ${\mathrm{NO}}_{3}^{-}-N$ . Increased biofilm thickness can lead to adverse effects on denitrification abilities of MBfRs *via* reduced substrate transfer efficiency that in turn affected ${\mathrm{NO}}_{3}^{-}-N$ removal rates. The effects of different pH values on MBfR nitrate removal were also evaluated (Figure 3C). The nitrogen removal capacity of MBfRs clearly first increased and then decreased with increasing pH. Optimal conditions were obtained by using the Design Expert software package, suggesting optimal a biofilm thickness of 653.31 μm, hydrogen pressure of 0.05 MPa, and pH of 7.78. Specifically, the model predicted a ${\mathrm{NO}}_{3}^{-}-N$ removal rate of 97.21%. The experimental conditions determined by the quadratic regression model were repeated three times to verify the reliability of the response surface model, while the average value of the total nitrogen removal rate obtained among the three experiments was 97.05% and the ${\mathrm{NO}}_{3}^{-}-N$ removal flux reached 1.15 g·m^−2^ d^−1^. These values were essentially consistent with the theoretical prediction of the model and thus, the optimal ${\mathrm{NO}}_{3}^{-}-N$ removal efficiency value obtained by RSM optimization is highly reliable and carries practical reference value.

### Microbial Community Structure Analysis

To investigate the temporal dynamics of microbial communities in the MBfRs, the ten most abundant taxonomic groups at the genus and phylum levels were investigated among different phases (i.e., before RSM optimization phase A1 and at stable operation phase A2). A total of 181,527 16S rRNA gene sequences were generated in this study (94,766 for A1 and 84,761 for A2) by using Illumina-MiSeq sequencing. A total of 1,511 OTUs were generated from the sequences at the 97% nucleotide similarity level, and the samples collected from sample A2 were grouped, respectively, into 745 OTUs, among which 421 species were coincident. This suggested that the microbial species in the two samples were different. However, the number of unique OTU in the sample A1 was 766, and the number of unique OTU in the sample A2 was 324. At stable operation phase the species of microorganism on PVC hollow fiber membrane surface reduced, which indicated the bacteria associated with these OTUs may play an important role in the deepening of membrane surface calcification phenomenon.

**Figure 4:**
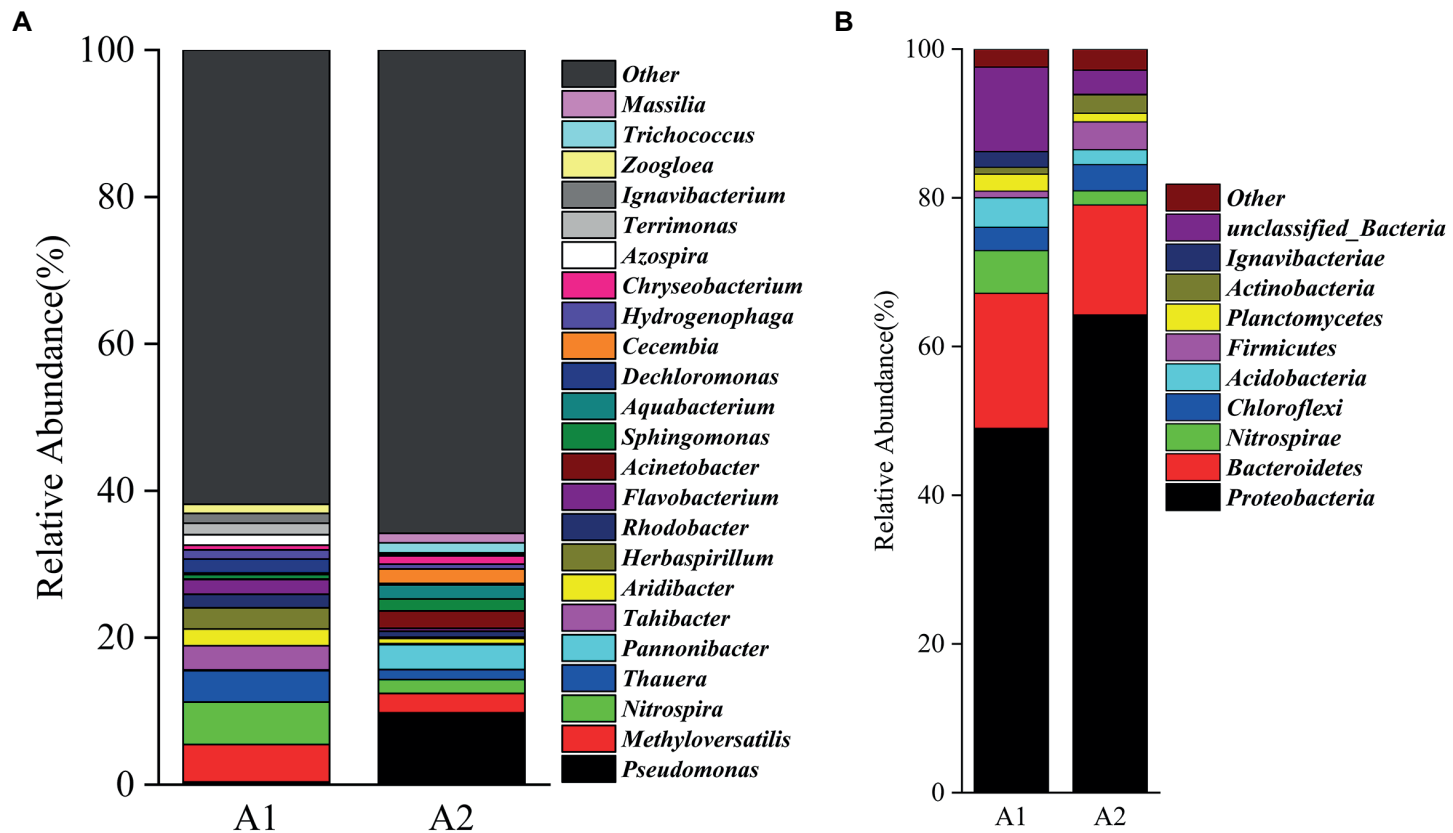
Taxonomic classification of 16S rRNA gene sequences in the A1 and A2 samples from the PVC surfaces of MBfRs classified at the **(A)** genus and **(B)** phylum levels.

*Proteobacteria*, *Bacteroidetes*, *Nitrospirae*, and *Chloroflexi* were the dominant phyla in the bacterial communities (Figure 4B), accounting for > 75% of the A1 and A2 communities. The most abundant phyla associated with nitrogen removal in the MBfRs were the *Proteobacteria*, *Chloroflexi*, and *Bacteroidetes* (Chang et al., 2022), with relative abundances of 64.24, 14.80, 1.89, and 3.51% in A2 during the stable operation phase. *Proteobacteria* (abundances from 48.99 to 64.23%) have been previously shown as prominent during nitrate removal (Su et al., 2020). Moreover, the enrichment of *Proteobacteria* has been associated with removal of nitrogen pollutants and in key roles in removing total nitrogen (Lee et al., 2013). *Bacteroidetes* are another important phylum associated with wastewater biological treatment systems and have also been reported in typical association with denitrifying during denitrification (Zhou et al., 2022). In this study, *Bacteroidetes* were the second most abundant genus, with relative abundances > 14% in both A1 and A2. Notably, the same predominant phyla were observed in A1 and A2, suggesting that optimization of the RSM denitrification performance of the MBfR did not affect the core microbiome of the reactor at the phylum level. Other, low relative abundance phyla were detected in the communities including *Nitrospira*e, *Acidobacteria*, *Firmicutes*, and *Planctomycetes*. Although these groups contributed little to the overall communities of A1 and A2, the entire communities nevertheless were likely involved in the improved hydrogen autotrophic denitrification performance of the MBfRs.

A total of 23 genera (relative abundance ≥ 1% in at least one sample) were identified in the two communities (Figure 4A). *Methyloversatilis* have been previously reported as denitrification bacteria (Li et al., 2019) and play a dominant role in ${\mathrm{NO}}_{3}^{-}$ removal in autotrophic reactors (Gao et al., 2015), accounting for 5.10 and 2.63% of the A1 and A2 communities, respectively. Comparison of the microbial communities of the A1 and A2 communities revealed that the microbial community structures of the membrane surface samples (A1 and A2) differed after RSM optimization. *Nitrosomonas*, which belong to ammonia-oxidizing bacteria, its proportions decreased from 5.75 to 1.87% of the communities, while anaerobic denitrification bacteria appeared, including *Pseudomonas* (12.75%), as previously described (Chang et al., 2022). These results suggested that the MBfR gradually became an anaerobic environment during the stable operation phase, thereby severely inhibiting the growth of nitrifying bacteria and providing optimal conditions for the growth of hydrogen autotrophic denitrifying bacteria. Notably, *Pseudomonas* relative abundances on the membrane surface of the two samples increased from 0.36 to 9.77%. *Pseudomonas* is a hydrogen autotrophic denitrifier and a nitrate-reducing taxa that has been shown to play a key role in hydrogen autotrophic denitrification during ${\mathrm{NO}}_{3}^{-}$ removal (Zhang et al., 2015). *Hydrogenophaga* is a well-known, but uncultured, hydrogen-oxidizing bacterial genus that characteristically reduces ${\mathrm{NO}}_{3}^{-}$ and can play a large role in H_2_-based MBfR functioning (Jiang et al., 2020). In addition, *Thauera* have been previously considered the most active denitrifying bacteria in sewage treatment systems, and were the primary contributor to nitrogen wastewater denitrification in a previous study (Dong et al., 2021). Thus, *Thauera* can likely improve optimized MBfR denitrification performance. Overall, these results suggested that the microbial community structures of the biofilm considerably changed compared to the phase before RSM optimization, due to significant influences by MBfR operation and optimization.

### Characterization of Calcification

Further analysis of our stably operating reactor revealed that biofilm formation was closely associated with Ca^2+^ concentration dynamics. Ca^2+^ has been previously observed as a common cation in water bodies that is associated with increased density of activated sludge and settling in activated sludge water treatment used in denitrification, while also simultaneously altering biofilm structures and affecting biofilm shedding (Liu et al., 2010). Calcification is more obvious in MBfRs operating for long periods of time, coinciding with increased Ca^2+^ deposits on biofilm surface and increased biofilm thickness on the hollow fiber membrane surfaces, causing more easy shedding. Slight calcification is helpful for biofilm formation, but severe calcification (which can be demonstrated by the thickness of the calcified layer) is detrimental to the growth of biofilm. Too thick biofilm will cause it to fall off more easily under the effect of hydraulic impact and vapor corrosion, resulting in lower denitrification efficiency. Consequently, the effects of calcification were characterized on the surfaces of the PVC hollow fiber membrane filaments in addition to the degree of Ca^2+^ enrichment by hollow fiber membrane filaments in MBfRs operating under stable anaerobic conditions.

#### SEM Characterization

**Figure 5:**
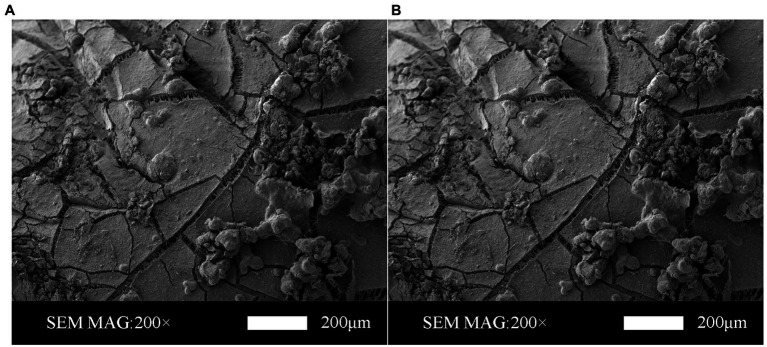
Scanning electron microscopy (SEM) images of PVC hollow fiber membrane surfaces in the stable operation phase. Magnification is shown at **(A)** 200× and **(B)** 500×.

Scanning electron microscopy was used to characterize the surface morphology of the PVC hollow fiber membranes (Figure 5). SEM analysis under various magnification levels indicated the presence of “particle”-like structures of the PVC hollow fiber membrane that were clearly visible on the surface during the stable operation phase (Figures 5A,B). Previous studies have shown that the surface structure of the PVC hollow fiber membrane in the early stage of the MBfR operation is mainly dominated by small pores and these membrane pores function as transport channels for hydrogen diffusion into biofilms in a bubble-free form (Nerenberg, 2016), while the membrane surface remains smooth without the presence of calcification.

Although the ${\mathrm{NO}}_{3}^{-}-N$ removal in MBfRs were improved in the stable operation stage, while the accumulated organic substances (Meng et al., 2010) were caused by the deposition of biopolymers on the membrane pores and the formation of gelatinous substances on the membrane surface that could enhance bacterial aggregation or binding to the surface. In addition, certain cations and anions were present in the MBfR, potentially leading to the phenomenon of concentration polarization on the surface of the PVC hollow fiber membrane that could lead to increases in salt concentrations on the membrane surface. Consequently, when the salt concentration exceeded the saturation concentration, chemical precipitation originating from inorganic crystals would occur on the membrane surface, leading to calcification. SEM visualizations revealed the presence of different shapes and sizes that can be clearly observed on the membrane surface, in addition to unique “particle”-like structures that were covered by inorganic calcium precipitates and the presence of particulates of different shapes and sizes due to calcification of the membrane surface. This then resulted in a disordered porous structure that would allow a mixture of calcium precipitation and microbial binding to the membrane surface, leading to increased membrane thickness and an increase in the weight of the membrane, leading to easier peeling.

#### AFM and XPS Characterization

**Figure 6:**
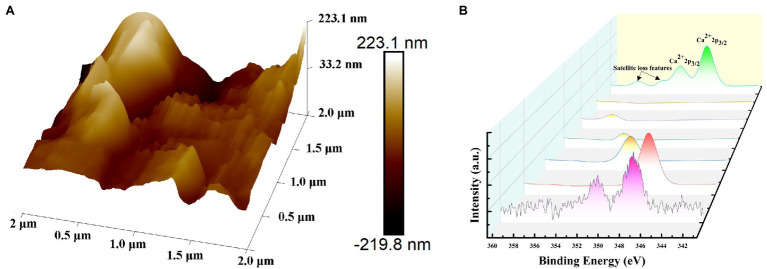
Images of the PVC hollow fiber membrane surfaces showing Ca levels after operation during the stable phase. Showed in **(A)** Atomic force microscopy (AFM) and **(B)** X-ray photoelectron spectroscopy (XPS).

Biofilm material roughness of PVC hollow fiber membrane surfaces was measured during the stable operation phase with AFM analysis and is shown in three-dimensional imaging over an area of 10 μm × 10 μm (Figure 6A). Membrane surface roughness magnitude can indicate calcium ion adsorption on the membrane surface to some extent, wherein greater surface roughness suggests more ready inorganic precipitation of calcium ions adsorbed onto the membrane surface. Concomitantly, the thickness of the membrane is not easily controlled, resulting in poor denitrification efficiency and thereby triggering calcification. Surface morphology was characterized using the peak force mode of the Bruker AFM instrument and the AFM images and roughness parameters were identified by using the NanoScope Analysis 1.9 software package (Bruker).

The surfaces were rougher in the stable operation phase and exhibited a higher degree of biofilm layering (Figure 6A). Roughness parameters including average roughness (*R_a_*), root mean squared roughness (*R_q_*), and the maximum height between the top and the bottom (*R*_max_) were used to quantitatively evaluate the surface properties of the PVC samples (Zhang et al., 2020). The *R_q_* values differed among membranes (57.8 nm for stable operation phase samples), wherein roughest surfaces corresponded to the largest roughness values. The *R_a_* also coincided with calcification, with a *R_a_* reaching 42.8 nm during the stable operation phase and the *R*_max_ of the surface reaching 414 nm in the same phase. Sludge, microorganisms, and other substances adhere to PVC hollow fiber membrane surfaces, leading to clogging of membrane pores and adhesion of inorganic substances such as calcium ions, thereby accelerating the formation of the calcification layer on the membrane surface.

X-ray photoelectron spectroscopy technology was employed to characterize the effects of calcification during stable operation and assess the presence of Ca on the membrane surface showed in (Figure 6B). The XPS pattern of the reactor during stable operation was compared against the standard binding energy spectrum, revealing that the Ca 2p was split into three characteristic peaks. Among these, the peak areas of the Ca 2p1/2 and Ca 2p3/2 orbitals were 1:2, and the spin energy interval was 3.55 eV, which was consistent with the standard energy spectrum. In addition, an additional characteristic peak was observed at 353.4 eV that further confirmed the observed increased calcium material on the membrane surface.

#### SEM-EDS Characterization

**Figure 7:**
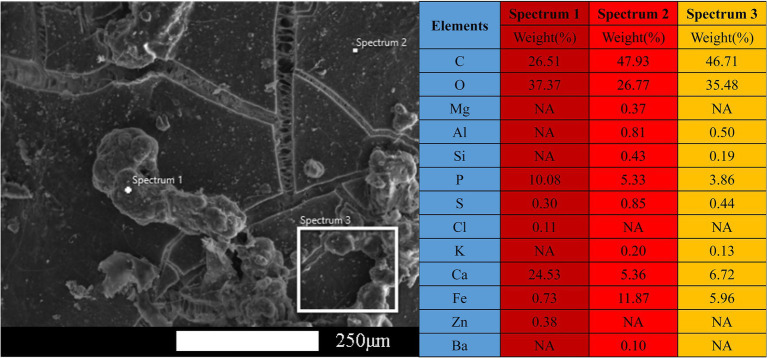
Scanning electron microscopy (SEM) morphology of the polyvinyl chloride (PVC) sample surface taken during stable operation, in addition to the point sweep energy spectra for spectrum 1, spectrum 2, and spectrum 3.

**Figure 8:**
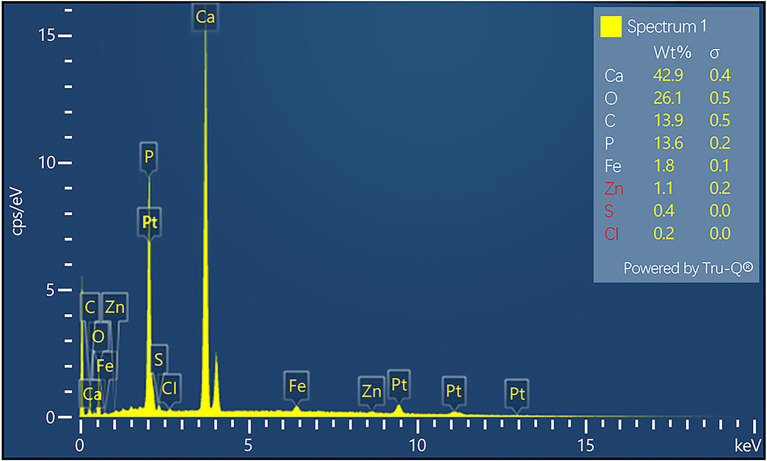
Scanning electron microscopy–energy dispersive spectrometer (SEM–EDS) characterization of the elemental distribution of spectrum 1 of the polyvinyl chloride (PVC) sample surface during the stable operation phase.

The surface morphology and elemental composition of the PVC samples during the stable operation phase are shown in Figure 7. Larger sized particles were observed forming channels and adhering to the sample surface that were likely due to the scratching of the sample on the PVC hollow fiber membrane surfaces. The energy spectrum points sweep of the particles attached to the surface revealed that the main elemental compositions were Ca, O, C, P, and Fe (spectrum 1 in Figures 7, 8). Further exploration of the elemental composition observed in spectrum 1 revealed that the primary elemental composition of spectrum 1 comprised Ca, O, C, and P, while Ca content was low and the relative percentage of Ca in the area of spectrum 4 was 42.9 weight % (*wt*.%). These results were consistent with the continuous enrichment of calcium on the PVC hollow fiber membrane during operation, resulting in increased calcium precipitation owing to the adhesion of inorganic substances like calcium to the membrane surface. The high content of Ca^2+^ and other cations led to an increase in inorganic substances in the reactor, resulting in a bridging effect between the cations and the substances attached to the membrane surface, thereby increasing the density of the membrane layer. Concomitantly, Ca^2+^ combined with specific acidic functional groups (e.g., R–COOH) can lead to the formation of complexes or gel layers, thereby further thickening the calcified layer and exacerbating the reduced membrane flux, leading to decreased membrane flux and reduced membrane life (Meng et al., 2010). PVC hollow fiber is a polymeric compound constructed from vinyl chloride that does not contain calcium, despite that calcium is the most abundant element on the surface of PVC hollow fibers during stable operation, which further confirming the presence of calcium-containing monomers or compounds on PVC hollow fiber surfaces. In addition to Ca, other elements were present based on the analyses including O, C, P, and Fe at *wt*.% of 26.10, 13.90, 13.60, and 1.80%, respectively. Thus, other trace impurity elements can be introduced during sample preparation and neglected when evaluating reactor performance. Overall, the primary elemental composition of the PVC hollow fiber membrane surfaces during stable operation phase comprised Ca, O, C, P, and Fe, with particularly high Ca^2+^ concentrations reflecting calcium precipitation on membrane surfaces and clear calcification over time.

#### XRD Characterization

**Figure 9:**
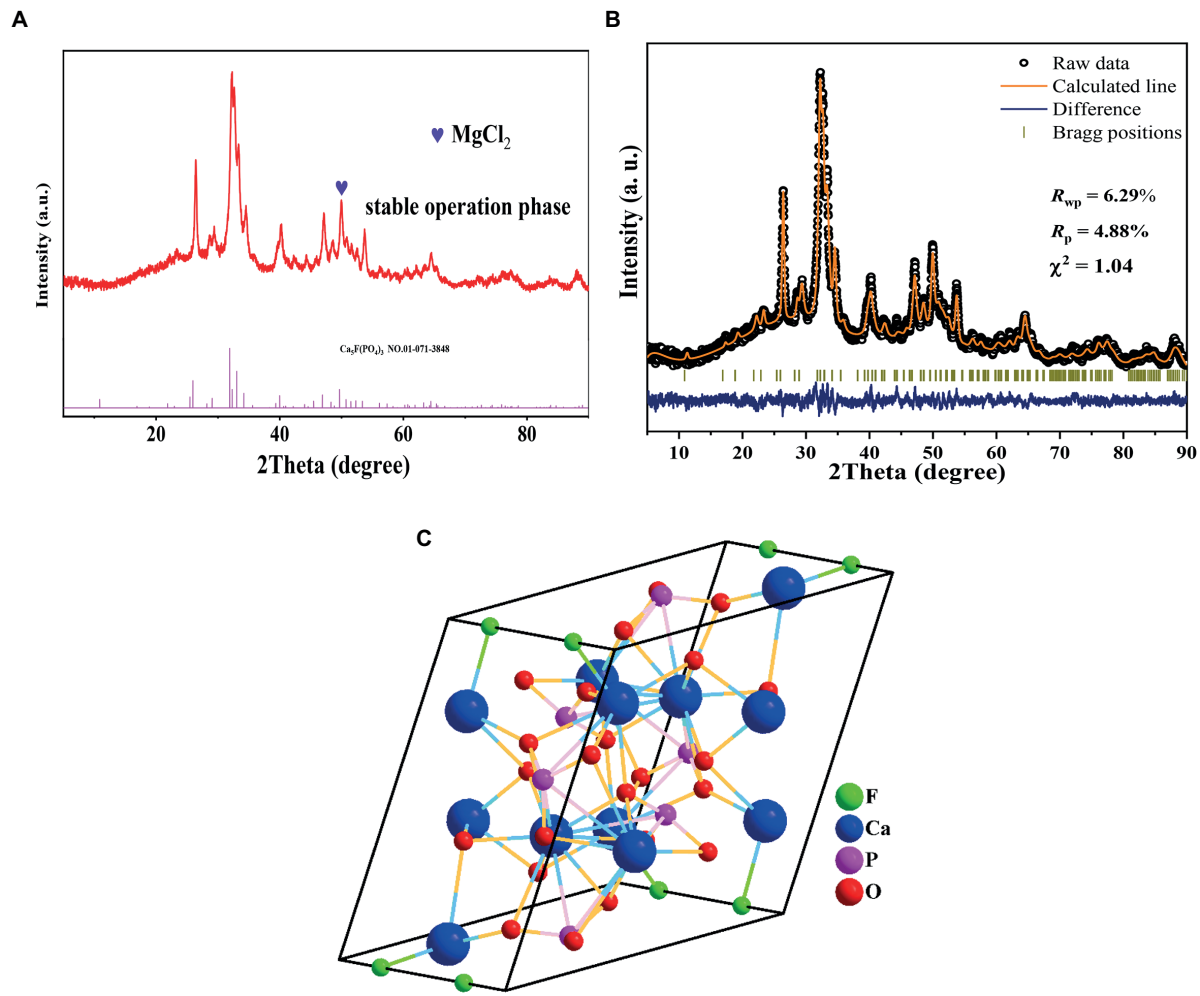
**(A)** X-ray diffraction (XRD) analysis of polyvinyl chloride (PVC) membrane sample surface during stable operation. **(B)** XRD refinement of PVC membrane sample surfaces during stable operation. **(C)** Crystal structure of Ca_5_F(PO_4_)_3_.

XRD patterns for PVC membrane sample surfaces were also analyzed during the stable operation phase (Figure 9A). Several obvious diffraction peaks were observed from surface samples in the range of 25–70°, although XRD curves also revealed bulges in the range of 20–40° into the back-bottom elevation. The overall diffraction peak position of the PVC sample was similar to that of Ca_5_F(PO_4_)_3_ (PDF#01–071-3,848) based on comparison in MDI Jade 9. XRD refinement of the data and crystal structures of Ca_5_F(PO_4_)_3_ (Figures 9B,C) indicated the presence of this compound in the optimized sample. Some of the diffraction peaks were compatible with MgCl_2_ (PDF#70–2,746) that is involved in conducting the treatment, although multiple impurity peaks remained without suitable compounds corresponding to them. These results followed from the PVC membrane surface carrying significant amounts of impurity plugging due to uninterrupted inlet water present during the experimental process. This results in membrane pore blockage and calcium-containing inorganic substances adhesion, thereby leading to altered diffraction peaks of PVC and calcification development. These dynamics could be due to inorganic substances comprising impurities such as Ca, Mg, and their compounds being present in the feed water (Meng et al., 2010), in addition to the presence of harmful substances like P being present (as verified with SEM–EDS). These substances were attached to the calcified layer on the membrane surface, leading to impurities remaining in the PVC membrane pores that produce complex diffraction peaks.

## Conclusion

In this study, a quadratic polynomial regression model of biofilm thickness, hydrogen pressure, and pH was established through response surface analysis based on single-factor experiments. Modeling led to observed response surface conditions of the reactor exhibited the ${\mathrm{NO}}_{3}^{-}-N$ removal flux was 1.15 g·m^−2^ d^−1^ when biofilm thickness was 653.31 μm, the hydrogen pressure was 0.05 MPa, and pH was 7.78. The model predicted the ${\mathrm{NO}}_{3}^{-}-N$ removal rate with an accuracy of 97.21%. In addition,, obvious calcium precipitation increasing after long-term operation which provide suitable habitat for biofilm formation. However, the thicker calcium precipitation easily led to the tendency of the biofilm to fall off easily due to cavitation. During stable operation, SEM, XPS, and SEM–EDS analyses of the PVC hollow fiber membrane revealed a disordered porous structure with significant calcium precipitation and calcification leading to the blockage of membrane pores. Characterization of samples from the stable operation phase indicated the presence of elements such as Ca, O, C, P, and Fe. Further XRD characterization confirmed that Ca_5_F(PO_4_)_3_ was added to the surface of the membrane filaments during the stable operation phase, in addition to the presence of other Ca and Mg elements and compounds. These results confirmed the presence of calcification on membrane surfaces during MBfR operation.

## Data Availability Statement

The original contributions presented in the study are included in the article/Supplementary Material, further inquiries can be directed to the corresponding author.

## Author Contributions

KD, XF, DW, and HaL: conceptualization and methodology. YY, KD, ZZ, and XF: project administration and data curation. HuL and KD: writing—original draft. KD and XF: writing—review and editing. KD, XF, YY, XZ, and HaL: supervision. All authors contributed to the article and approved the submitted version.

## Funding

This study was funded by Guangxi Natural Science Foundation (grant number 2022GXNSFFA035033), The Guangxi Key Laboratory of Theory and Technology for Environmental Pollution Control (grant number Guikeneng 2001K008), the Natural Science Foundation of China (grant numbers 51878197, 51978188), the Basic Ability Enhancement Program for Young and Middle-aged Teachers of Guangxi (grant number 2021KY0265) and Innovation Project of Guangxi Graduate Education (grant number YCBZ2022117).

## Conflict of Interest

The authors declare that the research was conducted in the absence of any commercial or financial relationships that could be construed as a potential conflict of interest.

## Publisher’s Note

All claims expressed in this article are solely those of the authors and do not necessarily represent those of their affiliated organizations, or those of the publisher, the editors and the reviewers. Any product that may be evaluated in this article, or claim that may be made by its manufacturer, is not guaranteed or endorsed by the publisher.

## Supplementary Material

The Supplementary Material for this article can be found online at: https://www.frontiersin.org/articles/10.3389/fmicb.2022.924084/full#supplementary-material
