# Supplementary figures and images for: Nitrogen Removal From Nitrate-Containing Wastewaters in Hydrogen-Based Membrane Biofilm Reactors via Hydrogen Autotrophic Denitrification: Biofilm Structure, Microbial Community and Optimization Strategies
Source: Front Microbiol. 2022 Jun 2;13:924084. doi: 10.3389/fmicb.2022.924084 (PMC9201494; doi:10.3389/fmicb.2022.924084)

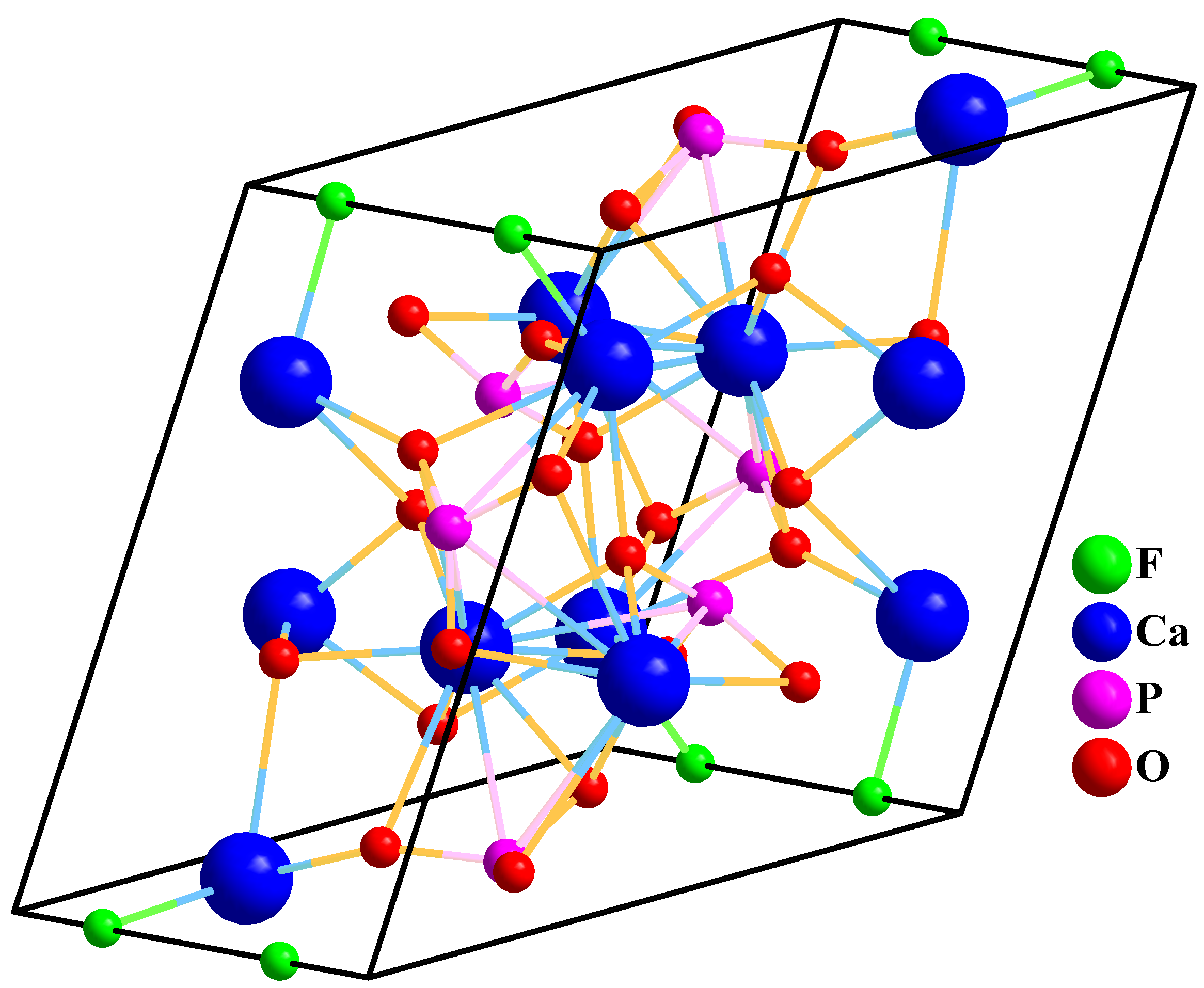

Supplement: Supplementary file 1 [file Image_1.TIF]
