# Supplementary material for: Nitrogen Removal From Nitrate-Containing Wastewaters in Hydrogen-Based Membrane Biofilm Reactors via Hydrogen Autotrophic Denitrification: Biofilm Structure, Microbial Community and Optimization Strategies
Source: Front Microbiol. 2022 Jun 2;13:924084. doi: 10.3389/fmicb.2022.924084 (PMC9201494; doi:10.3389/fmicb.2022.924084)
Supplement: Supplementary file 4 [file Table_1.DOCX]

To evaluate the factors that significantly influence the removal rate of nitrate, experiments were conducted for removing nitrate on the basis of the Box-Behnken design. The single-factor level experiments indicated that preliminary concentrations of biofilm thickness were estimated at 600, 650, and 700 μm, while hydrogen pressures were estimated at 0.02, 0.04, and 0.06 mg/L, and pH as 6.5, 7.5, and 8.5. The low and high levels for each factor were coded as shown respectively in Table 2. A second-order polynomial equation model was obtained by fitting regression analysis to the experimental data as follows:

$\boldsymbol{Y=96.08+1.35}\boldsymbol{X}_{\boldsymbol{1}}\mathbf{+}\boldsymbol{4.47}\boldsymbol{X}_{\boldsymbol{2}}\mathbf{+}\boldsymbol{3.31}\boldsymbol{X}_{\boldsymbol{3}}\boldsymbol{-3.86}\boldsymbol{X}_{\boldsymbol{1}}\boldsymbol{X}_{\boldsymbol{2}}$ (c) $\boldsymbol{+1.61}\boldsymbol{X}_{\boldsymbol{1}}\boldsymbol{X}_{\boldsymbol{3}}\boldsymbol{+2.96}\boldsymbol{X}_{\boldsymbol{2}}\boldsymbol{X}_{\boldsymbol{3}}\boldsymbol{-5.66}\boldsymbol{X}_{\boldsymbol{1}}^{\boldsymbol{2}}\boldsymbol{-9.23}\boldsymbol{X}_{\boldsymbol{2}}^{\boldsymbol{2}}\boldsymbol{-7.44}\boldsymbol{X}_{\boldsymbol{3}}^{\boldsymbol{2}}$

Y is the predicted nitrate removal rate and X_1_, X_2_, and X_3_ are the coded values for biofilm thickness, hydrogen pressure, and pH, respectively. The experimental data were compared with an ANOVA and evaluated based on lack of fit, pure error, adjusted regression coefficients (*R*2 adj), and predicted (*R*2 pred) regression coefficients (Table 3). The F-value obtained from an F-test was 78.61 and the *p*-value was < 0.0001, demonstrating that the model was adequate. In addition, a < 0.01% probability was observed that the model *F*-value could occur due to noise. The difference between *R*2 adj and *R*2 pred was < 0.2, also suggesting the applicability of the model. *p*-values are typically used to evaluate the significance of each of the coefficients and the effects of the interactions between optimal variables, wherein smaller *p*-values indicate more significant corresponding coefficients. The *p*-values of *AC*, *AB*, *BC*, *A*^2^, *B*^2^, and *C*^2^ were all < 0.05, indicating that factors *A*, *B*, and *C*, the squared terms *A*^2^, *B*^2^, and *C*^2^, in addition to the interaction effects *AC*, *AB*, and *BC* were significant model terms and the main contributors to nitrogen removal efficiency in the MBfR. Further, the adequate precision = 24.861 (i.e., the signal-to-noise ratio indicator of the model measurement) was > 4, indicating that the proposed model could be applied for the system. The *p*-value for the lack of fit was 0.1965 (e.g., > 0.05), while the coefficient of variation of Y (CV%) of the model was very low (1.51), indicating a very good model fit and credibility. Moreover, the experimental error was small, indicating that the model was accurate, credible, and can be used over a certain experimental range for detecting indicators. In addition, the coefficients of *A*, *B*, and *C* were equally important factors for nitrate removal from aqueous solution in the MBfR. Finally, the regression coefficient (*R*^2^) was 0.9902 and the adjusted regression coefficient was 0.9776, indicating a high significance for the model fit.

**Table 2.** Levels of each variable and corresponding nitrate removal rate obtained using an experimental Box–Behnken design.

| Run | Coded variable level | | | Real variable level | | | Nitrate removal rate (mg/L) | |
| --- | --- | --- | --- | --- | --- | --- | --- | --- |
|  | X_1_ | X_2_ | X_3_ | Biofilm thickness (μm) | Hydrogen pressure (MPa) | pH | Predicted | Experimental |
| 1 | 0 | 0 | 0 | 700 | 0.02 | 7.5 | 80.92 | 79.87±0.12 |
| 2 | 1 | −1 | 0 | 600 | 0.06 | 7.5 | 89.17 | 88.14±0.18 |
| 3 | −1 | 1 | 0 | 600 | 0.02 | 7.5 | 71.20 | 72.21±0.16 |
| 4 | 0 | 0 | 0 | 650 | 0.06 | 8.5 | 88.87 | 89.38±0.23 |
| 5 | 0 | 0 | 0 | 650 | 0.04 | 7.5 | 97.31 | 96.32±0.25 |
| 6 | −1 | 0 | −1 | 650 | 0.06 | 6.5 | 77.59 | 78.54±0.22 |
| 7 | −1 | 0 | 1 | 650 | 0.04 | 7.5 | 96.03 | 95.11±0.23 |
| 8 | −1 | −1 | 0 | 650 | 0.04 | 7.5 | 95.61 | 94.41±0.15 |
| 9 | 0 | −1 | −1 | 700 | 0.06 | 7.5 | 83.45 | 84.05±0.27 |
| 10 | 0 | 1 | 1 | 700 | 0.04 | 8.5 | 90.23 | 89.40±0.23 |
| 11 | 0 | −1 | 1 | 650 | 0.02 | 6.5 | 75.87 | 76.24±0.13 |
| 12 | 1 | 1 | 0 | 600 | 0.04 | 6.5 | 78.93 | 78.19±0.19 |
| 13 | 1 | 0 | 1 | 600 | 0.04 | 8.5 | 83.61 | 82.20±0.26 |
| 14 | 0 | 1 | −1 | 700 | 0.04 | 6.5 | 79.12 | 78.08±0.14 |
| 15 | 0 | 0 | 0 | 650 | 0.02 | 8.5 | 75.30 | 73.50±0.25 |
| 16 | 1 | 0 | −1 | 650 | 0.04 | 7.5 | 96.72 | 95.25±0.29 |
| 17 | 0 | 0 | 0 | 650 | 0.04 | 7.5 | 94.71 | 93.86±0.33 |

**Table 3.** ANOVA results for the quadratic model.

| Source | Sums of squares | df | Mean square | *F* value | *p*-value |  |
| --- | --- | --- | --- | --- | --- | --- |
| Model | 1,175.23 | 9 | 130.58 | 78.61 | <0.0001^a^ | Significant |
| *A*-biofilm thickness | 14.61 | 1 | 14.61 | 8.79 | 0.0209^a^ |  |
| *B*-H_2_ pressure | 160.12 | 1 | 160.12 | 96.38 | <0.0001^a^ |  |
| *C*-pH | 87.78 | 1 | 87.78 | 52.84 | 0.0002^a^ |  |
| *AB* | 59.60 | 1 | 59.60 | 35.88 | 0.0005^a^ |  |
| *AC* | 10.34 | 1 | 10.34 | 6.22 | 0.0413 ^a^ |  |
| *BC* | 35.11 | 1 | 35.11 | 21.13 | 0.0025^a^ |  |
| *A*^2^ | 135.03 | 1 | 135.03 | 81.28 | <0.0001^a^ |  |
| *B*^2^ | 358.55 | 1 | 358.55 | 215.84 | <0.0001^a^ |  |
| *C*^2^ | 233.10 | 1 | 100.21 | 140.32 | <0.0001^a^ |  |
| Residual | 11.63 | 7 | 1.66 |  |  |  |
| Lack of fit | 7.61 | 3 | 2.54 | 2.52 | 0.1965 | Not significant |
| Pure error | 4.02 | 4 | 1.01 |  |  |  |
| Cor total | 1,186.86 | 16 |  |  |  |  |
| *R*^2^ | *R*^2^=0.9902  *R*2 adj=0.9776  *R*2 pred=0.8922  Adequate Precision (AP) =24.861 | | | | | |

^a^Significance determined at the 95% confidence level (*p* < 0.05).
